# Is computer aided detection (CAD) cost effective in screening mammography? A model based on the CADET II study

**DOI:** 10.1186/1472-6963-11-11

**Published:** 2011-01-17

**Authors:** Carla Guerriero, Maureen GC Gillan, John Cairns, Matthew G Wallis, Fiona J Gilbert

**Affiliations:** 1 Health Service Research and Policy Department, London School of Hygiene and Tropical Medicine, London, UK; 2 Aberdeen Biomedical Imaging Centre, Lilian Sutton Building, University of Aberdeen, Aberdeen, UK; 3 Cambridge Breast Unit and Biomedical Research Institute, Addenbrookes Hospital, Cambridge, UK

## Abstract

**Background:**

Single reading with computer aided detection (CAD) is an alternative to double reading for detecting cancer in screening mammograms. The aim of this study is to investigate whether the use of a single reader with CAD is more cost-effective than double reading.

**Methods:**

Based on data from the CADET II study, the cost-effectiveness of single reading with CAD versus double reading was measured in terms of cost per cancer detected. Cost (Pound (£), year 2007/08) of single reading with CAD versus double reading was estimated assuming a health and social service perspective and a 7 year time horizon. As the equipment cost varies according to the unit size a separate analysis was conducted for high, average and low volume screening units. One-way sensitivity analyses were performed by varying the reading time, equipment and assessment cost, recall rate and reader qualification.

**Results:**

CAD is cost increasing for all sizes of screening unit. The introduction of CAD is cost-increasing compared to double reading because the cost of CAD equipment, staff training and the higher assessment cost associated with CAD are greater than the saving in reading costs. The introduction of single reading with CAD, in place of double reading, would produce an additional cost of £227 and £253 per 1,000 women screened in high and average volume units respectively. In low volume screening units, the high cost of purchasing the equipment will results in an additional cost of £590 per 1,000 women screened.

One-way sensitivity analysis showed that the factors having the greatest effect on the cost-effectiveness of CAD with single reading compared with double reading were the reading time and the reader's professional qualification (radiologist versus advanced practitioner).

**Conclusions:**

Without improvements in CAD effectiveness (e.g. a decrease in the recall rate) CAD is unlikely to be a cost effective alternative to double reading for mammography screening in UK. This study provides updated estimates of CAD costs in a full-field digital system and assessment cost for women who are re-called after initial screening. However, the model is highly sensitive to various parameters e.g. reading time, reader qualification, and equipment cost.

## Background

In the UK the NHS Breast Screening Programme (NHSBSP) currently screens over 1.9 million women per year and over 15,000 cancers are detected [1]. Since the introduction of the NHSBSP it is estimated that more than 18 million sets of mammograms have been taken and approximately 100,000 cancers have been detected [2]. As 80% of breast cancer cases occur in women over 50, the NHSBSP currently invites women aged 50-70 years for mammography screening every three years. However, by 2012 the age range will be increased to include women aged 47 to 73[3]

A number of factors, such as the introduction of double reading of mammograms and the extension of the age range for screening, has created a dramatic increase in demand for mammography readers [3,4]. Currently, demand increases are being met through the training of non radiologist film readers. Trained radiographers and breast clinicians show similar performance compared with radiologists [5,6]. However, it has been observed that this solution would not cope with the expected increase in manpower demand and the difficulty in recruiting radiologists trained in mammographic interpretation. As a result, finding technological solutions to reduce the manpower required to read mammograms is important to both guarantee the sustainability of the screening programme and achieve the commitment of increasing the age range of the screened population.

The development of CAD systems for mammography raised the possibility that a single reader using CAD could match the performance achieved by double reading [7]. However, the clinical efficacy of CAD in mammography remains controversial. There have been conflicting reports and inconsistent estimates of its impact on cancer detection rates and recall rates, primarily due to limitations in study design and intrinsic study biases [8-10]. A recent Meta-analysis [11] concluded that, compared to single reading alone, single reading with CAD resulted in a significant increase in recall rate, but no significant improvement in cancer detection rate. Furthermore, double reading, compared to single reading, significantly increased the cancer detection rate, but reduced the recall rate, particularly when used with arbitration. A large prospective trial (CADET II) reported a comparable cancer detection rate for single reading with CAD compared to double reading but with a small but significant increase in recall rate [12]. Despite the potential advantage of reducing the manpower required, the use of CAD for a single reader has not been introduced in the NHSBSP. Most screening units still use film screen mammography and perform double reading [13,14]. Double reading with arbitration of discordant cases by an independent third reader provides the highest small cancer detection rate whilst maintaining low recall rates [13,14]. However, screening units are in the process of introducing digital mammography, which will facilitate the use CAD but there is still uncertainty about the incremental cost associated with the adoption of CAD technology [15].

The CADET II study, compared the diagnostic performance of single reading with CAD with double reading among a cohort of 31,057 women undergoing routine screening in England [12]. This study uses data from the CADET II trial, to estimate the cost effectiveness of screening with a single reader using CAD compared with double reading over a seven year follow-up period. In order to investigate how the cost effectiveness of CAD changes according to the number of women screened per unit, the analysis has been performed for low, average and high volume screening units separately.

## Methods

### Effectiveness

The effectiveness of single reading with CAD compared with double reading was taken from the CADET II trial. The study was approved by the South East Multi-Centre Research Ethics Committee and written informed consent was obtained from all participants.

In the trial, women attending routine screening in the NHSBSP were randomly assigned to have their mammograms read by double reading, single reading with CAD, or both (at a ratio of 1:1:28). The two main indicators of effectiveness used to estimate the cost effectiveness of single reading with CAD compared with double reading were: cancer detection rate and recall rate for further assessment. There were no significant differences in the cancer detection rates between single reading with CAD and double reading (7.02 and 7.06 per 1000 respectively) [12]. However, the recall rate was significantly lower for double reading (3.4%) compared with single reading with CAD (3.9%) [12]. Given that no differences in cancer detection rates were found, cost effectiveness was estimated in the baseline analysis in terms of cost per 1,000 women screened. Uncertainty around the incremental number of cancers detected with CAD versus double reading was explored in a probabilistic sensitivity analysis.

### Cost

In accordance with the National Institute of Health and Clinical Excellence recommendations, the analysis was performed using a health and social service perspective [16]. All costs are expressed in 2007/8 prices. The objective of this cost analysis was to investigate whether or not single reading with CAD is cost saving compared with double reading in the context of data from CADET II. On the one hand, introducing CAD into routine breast screening is accompanied by increases in training costs, equipment costs and additional cost arising from the higher recall rate; on the other hand, CAD would allow for cost saving in mammography reading time. Thus, the costs of single reading with CAD and double reading per 1000 women screened can be summarised as follows:

CA=1000*a+1000*δ*c

CB=1000*b+1000*μ*c

Where: *a *is the cost of single reading with CAD; *b *is the cost of double reading; δ is the recall rate following single reading with CAD; μ is the recall rate following double reading and *c *is the cost of recall for further assessment, which is assumed to be the same for both the options.

The incremental cost of single reading with CAD versus double reading is calculated with the following formula: IC_CAD _**= **1000*(*a-b*) + 1000*(δ - μ)*c. As the two options showed no significant difference in cancer detection rates, single reading with CAD will be a cost effective intervention if IC_CAD _< 0, if on the other hand, IC_CAD _> 0 single reading with CAD strategy would be cost increasing for the UK NHSBSP and double reading should remain the routine practice. This analysis does not take account of any costs arising from the need to involve another reader to arbitrate discordant cases since arbitration policy varies considerably between centres and is difficult to generalise.

### Screening cost of single reading with CAD

The cost of screening using a single reader with CAD has four elements: the cost of purchasing and upgrading CAD equipment, the maintenance cost of the equipment, the cost of training the personnel and the cost of reading. Since the cost of purchasing and maintaining CAD equipment depends on the number of women screened each year in individual screening units, the analysis has been conducted separately for high, average and low volume screening units. For this analysis a low volume unit is defined as a unit screening between 5,000 and 20,000 women aged 50-70 annually [17]. An average size screening unit is defined as a unit screening between 20,000 and 30,000 women annually. A high volume screening unit is defined as a unit screening between 30,000 and 40,000 women annually. Importantly, the analysis assumes that the unit is already digital or is going to be digital independently from any decision over the introduction of CAD [15].

### Reading cost

The overall reading cost depends on two main elements: the cost per minute of the personnel reading the film and the time spent reading the films. Due to the increasing number of women screened each year by the NHSBSP there is an increasing number of radiographers and breast clinicians involved in mammography screening. In the NHSBSP screening units, 53% of mammograms are read by radiologists and 47% are read by either breast clinicians or advanced practitioners [18]. The mean cost per hour (£71.35) of the staff involved in reading mammograms in UK was estimated using an average of the hourly cost of radiologist and breast clinician or advanced practitioner weighted by the proportion of radiologists and breast clinicians/advanced practitioners involved in reading mammograms in the UK. The cost per hour of those involved in reading mammograms was retrieved from the Agenda for Change pay band and the "Unit Costs of Health and Social Care 2008" study [19-21]. Both advanced practitioners and breast clinicians were assumed to be band 7 [21]. Radiologist cost per hour was assumed to be equal to the cost per hour of a medical consultant and was taken from Curtis (2008) [19]. According to the study conducted by Taylor et al [22], the average reading time per case weighted by number of cases is 58.5 seconds (0.975 minutes). The estimated reading cost per women screened using single reading with CAD was estimated to be £1.17.

### Training cost

According to the CADET II trial, staff required four days training in the use of CAD technology [12]. On the first day, the training aims to provide adequate information about software applications. On the second day, personnel are trained in how to use the software. On the third and fourth days, a bank of existing films is used to simulate routine mammography screening with CAD. In addition, it is assumed that personnel are retrained every three years for three hours. Using this information, the equivalent annual cost of training per reader was estimated over a seven year period using a 3.5% discount rate [23]. On average, a reader in England and Wales reads 5,800 cases per year (S Sellars, National Cancer Screening Programmes, personal communication). Thus, the mean cost of training per women screened, £0.02, was calculated dividing the equivalent annual cost of training a reader by the average number of cases read each year.

### Equipment cost

Based on data from the CADET II study, one CAD system would be able to process, at least, 10,000 analogue cases per year [12]. In order to account for this element of uncertainty it is assumed that in a low volume unit two workstations/systems would be required, in an average volume unit between two and three, and in a high volume unit between three and four workstations. Estimates of the cost of purchasing the CAD system(s) in the differently sized units were obtained from manufacturers assuming a bulk purchase for ten or more screening units. The equivalent annual cost of the equipment was estimated assuming a seven year lifespan, 3.5% discount rate and no scrap value [23]. The resulting average CAD equipment cost (SD) per case read is £0.65 (0.22), £0.41 (0.06) and £0.40 (0.03) for low, average and high volume sites respectively. According to the manufacturer, the software for the CAD system is likely to be upgraded every three years. The equivalent annual cost per women screened of upgrading CAD software was estimated to be £0.08 for both high and average volume sites and £0.11 for low volume sites.

### Maintenance cost

The maintenance cost of CAD equipment depends on the type of annual maintenance contract that the screening unit purchases. Assuming a comprehensive contract costing 6% of the cost of the system the resulting cost per women screened is £0.14, £0.15 and £0.22 in high, average and low volume sites respectively.

### Screening cost of double reading

The reading time per case for two readers reading four digital mammograms was estimated to be 1.95 minutes [22]. The cost per hour of the staff personnel involved in reading was the same of single reading with CAD. Thus, the resulting cost per case read using double reading was calculated to be £2.35.

### Assessment cost

Once a women is recalled for further assessment after initial screening there are several possible breast cancer diagnostic methods [24].

In general there are two mutually exclusive diagnostic categories to detect a breast cancer during the assessment visit: on clinical and/or radiological grounds only (very rare), or biopsy. 95% of cancers in UK are diagnosed non operatively by needle biopsy (cytology or core biopsy) and 5% require open biopsy [24] The simplest (and least expensive) assessment visit consists of an ultrasound (70% of cases) and/or repeated mammograms (43% of cases) [25].

If there is still uncertainty needle biopsy and/or an open biopsy is performed. According to the NHSBSP in England 32% of assessment visits include a needle biopsy (of these 29% are core biopsy, 2% core biopsy and cytology and 1% cytology only) while in 2.2% of visits women are referred for additional open biopsy) [24,26].

The unit cost of diagnostic imaging techniques is £83 and £75 for mammography and ultrasound respectively [27,19] A cost of £176 for core biopsy and £130 for cytology has been used in the analysis. These estimates have been obtained from Griebsh et al. and uplifted to 2008 prices [25,19]. Assuming that a person referred for needle biopsy was already imaged with both mammography and ultrasound and all patients undergoing open biopsy had a prior needle biopsy, by combining the above mentioned percentages the average cost of an assessment clinic is estimated to be £153 per women. This estimate is broadly similar to the £125 estimated by the NHS Purchasing and Supply Agency [28].

### Sensitivity analyses

To assess the robustness of the results to the assumptions made, extensive one-way sensitivity analyses were carried out separately for high and low volume screening units. The incremental cost of single reading with CAD versus double reading was estimated assuming both a 0.3 and 0.8 percentage point difference in recall rate between the two reading options [12].

The cost of an assessment visit depends on the type of diagnostic procedures that are performed. In order to estimate how variations in assessment cost influence CAD cost effectiveness, the incremental cost of CAD versus double reading was estimated assuming a price ranging from £75, if the women is referred for a repeated mammography, to £662, if an open biopsy is performed (in addition to a preliminary ultrasound/mammography and needle biopsy) [25-27] Further, one way sensitivity analyses estimated the incremental cost of single reading with CAD, assuming a range of reading times per reader (and no difference between single and double readers), different annual CAD maintenance cost and different mixes of readers (all radiologists and all advanced practitioners/breast clinicians).

### Probabilistic sensitivity analysis

Uncertainty around model parameters such as cancer detection rates with and without CAD (double reading), assessment cost and reading time was explored in a probabilistic sensitivity analysis. A Monte Carlo simulation was carried out using Excel. A distribution was assigned to all input parameters. According to Briggs et al. [29] a beta distribution was used for binomial data (e.g. probability of being re-called), while a Gamma distribution was assigned to cost values and resource use (e.g. reading time). Cost effectiveness acceptability curves (CEAC) were generated for the three sizes of screening unit. The cost; the benefit (incremental number of cancers detected) and the net benefit of the two strategies were calculated for 1,000 Monte Carlo simulations according to the following formula [29]:

Expected net benefit=λ(ΔE)−(ΔC)

Where λ is the willingness to pay per cancer detected, ΔE is the incremental number of cancers detected with CAD and ΔC is the incremental cost of the CAD strategy.

Each CEAC displays the probability that an intervention is cost effective for a range of willingness to pay values (£0-£50,000).

## Results

### Base Case Analysis

Table 1 shows the incremental cost per 1,000 women screened of single reading with CAD versus double reading for high, average and low volume screening sites. The reading cost constitutes the main difference between the two options. Substituting double reading with single reading with CAD would reduce the reading time by 0.975 minutes per case allowing for a cost saving of £1,176 per 1,000 women screened. The additional cost of purchasing and upgrading CAD equipment varied according to the screening volume of the unit. In low volume units, the incremental equipment and upgrading cost was estimated to be £764 per 1,000 women screened. This cost falls in high and average volume sites to £475 and £496 because the CAD price decreases if the number of workstations purchased by the screening unit increases. The maintenance cost, which is calculated according to the price of the CAD system, is also lower for both high and average volume units compared with low volume units (£144 and £148 versus £217).

**Table 1:** Base case result: difference in cost of single reading plus CAD versus double reading per 1000 women screened

Incremental cost single reading with CAD versus double reading			
	**High volume unit** **30,000-40,000** **per annum**	**Average volume unit** **20,000-30,000** **per annum**	**Low volume unit** **5000-20,000** **per annum**

Reading cost	-£1176	-£1176	-£1176

Equipment and pgrading cost	£475	£496	£764

Maintenance cost	£144	£148	£217

Training	£20	£20	£20

Assessment cost	£764	£764	£717

*Total incremental cost*	£227	£253	£590

The incremental assessment cost resulting from the higher recall rate of single reading with CAD versus double reading is £764 per 1,000 women.

In all three sizes of unit, single reading with CAD is thus cost increasing compared with double reading as the additional cost of purchasing, upgrading and maintaining the equipment, the cost of training and the higher assessment cost are greater than the savings arising from the lower reading cost. The incremental cost per 1,000 women screened with CAD versus double reading is £590 for low volume units and £253 and £227 in average and high volume units respectively.

### One Way Sensitivity Analyses

The results of one way sensitivity analyses for high, average and low volume units are summarised in Table 2.

**Table 2:** One way sensitvity analyses

Incremental cost single reading with CAD versus double reading			
	**High Volume Unit** **3000-4000** **per annum**	**Average Volume Unit** **20000-30000** **per annum**	**Low Volume Unit** **5000-20000** **per annum**

Reading cost (1.50 to 0.50 minutes)	-£402;£800	-£380;£825	-£43;£1162

Reader qualification (all radiologists to all advanced practitioners)	-£320;£849	-£295;£875	-£41;£1212

Cost of the equipment (£0.30 to £1)	£132;£831	£181;£841	£240;£977

Recall rate difference between CAD and double reading (3% to 8%)	-£76;£685	£-52;£711	£284;£1047

Assessment cost (£75 to £662)	-£186;£2896	-£161;£2976	£176;£3491

Maintenance cost (no cost to 10% the cost of the equipment)	£84;£323	£105-£387	£372-£735

As expected, the assessment cost, assumed to range between £75 and £662, has the greatest impact on CAD cost effectiveness. If an assessment visit were only to include additional mammography, CAD is always cost saving. If the cost of an assessment visit is higher than assumed, CAD is more markedly cost increasing compared to double reading.

Reading time per case, assumed to range between 0.50 minutes to 1.50 minutes, also influences the cost effectiveness of CAD. If the average reading time per case is 0.50 minutes, then introducing CAD in routine screening in the NHSBSP increases cost in all screening units (£800 high volume units, £825 average volume units and £1162 low volume units). Alternatively, if reading time per case is 1.50 minutes, then single reading with CAD produces a cost saving even in low volume units.

If all the readers are radiologists the potential savings arising from the introduction of CAD are for high, average, and low volume units £320, £295 and £41per 1,000 women screened. Single reading with CAD is cost increasing if all the readers are breast clinicians/advanced practitioners because of the lower cost of non-radiologist reading time per minute.

Holding other parameters constant, cost effectiveness was also found to be sensitive to the difference in recall rates between CAD and double reading. If the difference in recall rate is 0.3 percentage points, then CAD is cost saving in high and average volume units. As the difference in recall rates increases the incremental cost of CAD increases.

### Probabilistic Sensitivity analysis

**Figure 1:**
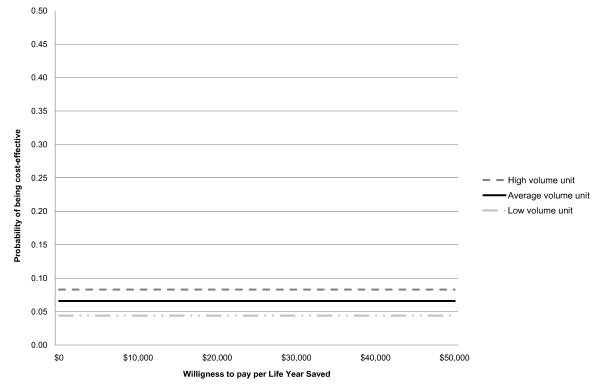
**Cost effectiveness acceptability curves for high, average and low volume units**.

Three cost effectiveness acceptability curves of CAD versus double reading - one for each screening volume - are shown in Figure 1. The curves plot the proportion of costs and effects ratios that are cost effective for a range of monetary values that the decision maker is willing to pay for a given health outcome improvement (in this case per cancer detected). As there is very little difference in cancer detection rates between CAD and no CAD (diff = -0.0044053 95% CI: -0.0739949, 0.0651843) over 1,000 simulations the average incremental number of cancers detected with CAD compared with double reading is effectively zero. CAD is cost effective only if it is a cost saving intervention (incremental cost of CAD is negative). Thus, as shown in Figure 1 independently from the amount the NHS is willing to pay to detect an additional cancer, CAD has a 8%, 7% and 4% probability of being cost effective in high, average and low volume units respectively.

## Discussion

The results of a large randomized trial conducted in the UK reported that a single reader using CAD technology would perform as well as two readers in detecting breast cancer in women aged between 50 and 70 [12]. Based on these data, this analysis examined the cost-effectiveness of using single reading with CAD compared with double reading in the NHSBSP. As with previous cost effectiveness analyses this study suggests that CAD might be a cost-increasing intervention compared with double reading in all sizes of screening units because the savings arising from the shorter CAD reading time will be offset by the cost of staff training, the cost of purchasing, upgrading and maintaining the CAD equipment, and in particular the increased cost of assessment. [5,30,31]. It has been estimated that introducing single reading with CAD would produce an additional cost of £227, £253 and £590 per 1,000 women screened in high, average and low volume units respectively.

The results of the one way sensitivity analyses show that the baseline incremental cost of single reading with CAD versus double reading was highly sensitive to model parameters assumed for all sizes of units. Assessment cost, reading time per case, reader qualification and the difference in recall rates were found to have the highest impact on the incremental cost of CAD. As expected, a longer reading time per case would increase the potential saving arising from the introduction of CAD and CAD would be cost effective in all types of units. Similarly, the higher the reader qualification (and thus the cost of reading per minute) the higher are the savings arising from CAD. It has yet to be determined, if CAD were introduced routinely, who will be allowed to read mammograms (only radiologists or radiologists and radiographers). Sensitivity analyses also suggests that if the difference in recall rate is as low as 0.3%, then CAD would be cost effective in both high and average volume units [12]. Probabilistic sensitivity analysis confirms the robustness of the study findings. When all parameters are varied, the probability of CAD being cost effective is 8% in high volume units, 7% in average volume units and only a 4% chance of being cost saving in low volume units.

If cost-effectiveness were to be measured in terms of cost per recall averted, double reading would dominate CAD in all the types of units (being less costly and more effective).

The present study improves the existing evidence on the cost effectiveness of CAD versus double reading in routine breast screening in UK. The effectiveness data used to populate the model were obtained from a large prospective randomised trial evaluating CAD versus double reading in UK breast screening units.

In December 2007, the UK Government's Cancer Reform Strategy made the commitment to replace film mammography with digital mammography [15]. Digital mammography has been shown to be a cost effective alternative to film mammography in UK and will progressively replace film screen mammography in the next few years [21]. This study evaluates, for the first time in the UK, the cost effectiveness of single reading with CAD against double reading assuming that screening units use digital mammography systems. As noted by Lindfors et al. [31], the cost effectiveness of CAD is heavily dependent of the cost of CAD technology and implementation of CAD in a digital mammography setting should be a much less labour intensive process and likely to increase its cost effectiveness.

In interpreting the results and findings, several limitations and assumptions should be considered. The study assumes that the effectiveness of CAD using a digital mammography system is the same as observed in the CADET II trial where film screen mammography was used. To date, there have been no prospective studies conducted on the effectiveness of CAD using a digital mammography system. Further research is needed to establish whether, following the introduction of digital mammography, single reading with CAD and double reading will continue to show similar effectiveness in cancer detection rates. Another limitation of this study is that the reading time is based on an examination of four digital images. When a woman is screened for the second time, the reading time per case may be longer if prior mammograms have to be viewed for comparison, thus the potential savings in reading cost by using single reading with CAD may have been underestimated [32,33]. However, the availability of previous round mammograms could make it easier for the reader, in the case of CAD, and readers, in the case of double reading, to decide and thus reduce average reading time. As a consequence, the potential savings in reading cost resulting from the introduction of CAD in routine screening are difficult to estimate. Although the unit cost of an assessment visit used in the present study has been estimated using recent data from NHSBSP it is subject to great uncertainty. Procedures for diagnosing breast cancer (and thus the costs of assessment) vary between regions. For example in Northern Ireland where there is still a high proportion of cancer diagnosed by cytology only (4%), the average cost of an assessment visit is likely to be lower than the national average [24]. In addition the costs of assessment are sensitive to procedural innovations. This analysis, for instance, has not taken account of the use of Vacuum Assisted Biopsy (VAB) devices. There is increasing evidence that VAB is a powerful tool to diagnose microcalcifications, to improve the diagnostic rate and to reduce under-staging. However, due to both the high cost and the high non operative diagnosis rate achieved by other sampling methods, the use of VAB has been limited in the NHSBSP [34]. If VAB were to be used in routine clinical care the cost of assessment and thus the incremental cost of CAD versus double reading would increase significantly.

In addition, the study does not distinguish between the reading time of radiologist and non-radiologist readers. Although no significant difference in reading time between these professional groups has been reported were there to be one the potential saving arising from CAD would have been underestimated [35]. The estimated price of purchasing and upgrading the CAD systems might be conservative. If CAD is introduced into routine practice in the screening programme the price of a CAD system and upgrading is likely to decrease due to bulk purchases and price negotiation with commercial suppliers. In addition, the number of CAD systems and workstations that screening centres would purchase is difficult to estimate since this is dependent on both screening volume and the number of readers.

There are a number of clinical issues associated with the use of CAD that should be addressed within the limitations and assumptions of this analysis.

It has been assumed that the lesion types recalled by single reading with CAD are similar to those with double reading. In CADET II, for cancer cases, there was no significant difference in recall rate between calcifications and masses [29].

However, there is no information on the suspected lesion type that prompted recall of non cancer cases (false positives), by either reading regimen. Thus assessment costs have been assumed to be comparable for both arms of the study. However, there is evidence that CAD has a greater sensitivity with respect to detection of microcalcification, particularly when used with digital mammography, that could result in higher recall rates and associated assessment costs [36]. Thus assessment costs for single reading with CAD may be underestimated. Recall after screening mammography has been associated with increased anxiety and stress that could also result in increased healthcare costs and contribute to an underestimate of the true costs of recall and assessment in the current analysis [37,38].

In addition, no account has been taken of any costs arising from the need to involve another reader to arbitrate discordant cases. In view of the high false marker rate with CAD, arbitrating all cases where there is discordance between the single reader and CAD would be equivalent to double reading with CAD and would not be cost effective [39]. If the NHSBSP introduced single reading with CAD, appropriate training to familiarise readers with the performance of CAD systems (should minimise the number of cases that would require arbitration by a third reader [40,41].

## Conclusion

The use of two readers to independently review screening mammograms has been shown to be a cost effective intervention to reduce mortality from breast cancer [33-42]. As single reading with CAD has been shown to achieve the same detection rates as two mammography readers in the CADET II study, this too could be cost effective, provided the recall rate is not greatly increased [12]. The results of this modelling study indicate that CAD is a cost-increasing strategy in all units regardless of screening volume because the cost of purchasing CAD equipment, and the incremental assessment cost outweighs the savings arising from the shorter reading time. However, it is acknowledged that the model is highly sensitive to changes in parameters e.g. reading time, reader qualification, and equipment cost. In addition, more research is needed to estimate CAD performance when used with digital mammography systems, and further information is also needed on the reading times of radiologists and advanced practitioners using CAD.

## List of abbreviations used

CAD: Computer aided detection; NHSBSP: National Health Service Breast Screening Programme

## Competing interests

The authors declare that they have no competing interests.

## Authors' contributions

CG and JC were responsible for the economic model, data analysis and interpretation, and drafting the manuscript. FJG, MGCG and MGW contributed to the study design, data collection, interpretation of results and reviewing of the manuscript. All authors read and approved the final version of the manuscript.

## Pre-publication history

The pre-publication history for this paper can be accessed here:

http://www.biomedcentral.com/1472-6963/11/11/prepub
